# Identification of immune-related cells and genes in the breast invasive carcinoma microenvironment

**DOI:** 10.18632/aging.203879

**Published:** 2022-02-04

**Authors:** Xingfeng Chen, Yujing Wang, Yue Li, Geli Liu, Kui Liao, Fangzhou Song

**Affiliations:** 1 Molecular Medicine and Cancer Research Center, Chongqing Medical University, Chongqing 400016, China; 2 Department of Clinical Medicine, Changde Vocational and Technical College, Changde, Hunan 415000, China; 3 School of Medical Information, Chongqing Medical University, Chongqing 400016, China; 4 Department of Radiotherapy, The First Affiliated Hospital of Chongqing Medical University, Chongqing 400016, China

**Keywords:** breast invasive carcinoma, tumor microenvironment, bioinformatics, prognosis

## Abstract

The clinical prognosis of breast cancer is closely related to its infiltrating immune status. The study sought to explore tumor-infiltrating immune cells (TILs) and immune-associated genes in the tumor microenvironment of breast invasive carcinoma (BRCA). The ESTIMATE algorithm was used to evaluate the microenvironment of breast cancer patients in TCGA database. The tumor's matrix score and immune score were obtained. The median was divided into two sub groups according to the median of the score, and the correlation between the score and prognosis was also discussed. Differentially expressed genes were screened from two subgroups with high and low score of breast cancer, and the differentially expressed genes were analyzed by GO and KEGG enrichment to explore their possible molecular functions, biological processes, cellular components and signal pathways involved in gene enrichment. It was found that there was a significant correlation between immune score and five-year survival rate, and the high score group had a better prognosis. Macrophage M1 and T cell CD8+ cells were positively related to 5-year overall survival in patients with breast invasive carcinoma. However, Macrophage M2 was negatively related to 5-year overall survival. We also observed that the low expression of four genes (CLEC3A, MCTS1, PDP1 and TCP1,) was related to favorable survival outcomes. High expression of FOXP3, CXCL9, CCR5, CXCR3, and CD37 was related to a high overall survival rate in BRCA. We identified a list of immune – related cells and genes that are useful for Prognostic evaluation and individualized treatment of BRCA.

## INTRODUCTION

Breast cancer has become the most common and most prevalent tumor among women worldwide. The incidence rate of breast cancer is increasing, and the incidence rate of death is decreasing. Although oncologists have conducted extensive and in-depth studies on the causes of breast cancer, the exact pathogenesis is unclear, and targeted prevention and treatment are difficult [1–3]. The presence of tumor specific immune responses in breast cancer has been suppressed, even in the early stages of cancer development. The microenvironment that inhibits the body’s immunity plays an important role in this process, which may be related to its effect on tumor antigen-specific immune cells [4–6]. The design of therapeutic regimen against immunosuppressive microenvironment is a new strategy for breast cancer immunotherapy. However, the molecular mechanism of immune suppression in the microenvironment of breast cancer is still unclear, which limits the development of specific targeted therapy [7–10].

Tumor microenvironment is a special microenvironment for tumor cells to survive and develop [11]. It is a complex whole composed of a variety of cells and extracellular matrix that affect the occurrence, development, invasion and metastasis of tumor. These components are collectively referred to as tumor microenvironment. In terms of its broad components, tumor microenvironment includes endothelial cells and their precursors, tumor associated fibroblasts, T lymphocytes, B lymphocytes, natural killer cells, antigen presenting cells (such as macrophages, dendritic cells), tumor associated macrophages, and extracellular matrix [12–14].

Tumor cell immune escape is one of the important characteristics of tumor, and it is also the main reason for the poor effect of conventional treatment. Human immunity, especially cellular immunity, is closely related to the occurrence and development of tumor. Tumor immune escape is a multi-link and multi-mechanism process, such as the immune modification of tumor cells, the immune tolerance of tumor patients and the decrease of tumor microenvironment immune function. Tumor microenvironment is a kind of microenvironment composed of a variety of stromal cells and cytokines around tumor cells, which is conducive to tumor growth. It is the first place for tumor antigen to contact with the body’s immune system, so it is of great significance in tumor immunology [15–18].

This study included 968 patients with breast cancer from the TCGA database. The ESTIMATE algorithm was used to evaluate the tumor microenvironment, and the matrix score and immune score of the tumor were obtained. According to the median score, the tumor was divided into two subgroups, high and low. The differentially expressed genes were screened for two subgroups related to the prognosis of patients, and go and KEGG enrichment analysis were performed for the obtained differentially expressed genes to explore their possible molecular functions, biological processes, cellular components of gene enrichment and signal pathways involved. For each of the differentially expressed genes, we drew the K-M survival curve, and screened out the differentially expressed genes significantly related to the five-year survival rate. In order to further explore the core regulatory genes, we constructed a protein interaction network for the survival related differentially expressed genes screened in the previous step, and carried out gene enrichment analysis and module mining. Finally, we screened the most significant prognostic related core differentially expressed genes from the differentially expressed genes.

## RESULTS

### Workflow of TCGA- breast invasive carcinoma data processing

**Figure 1:**
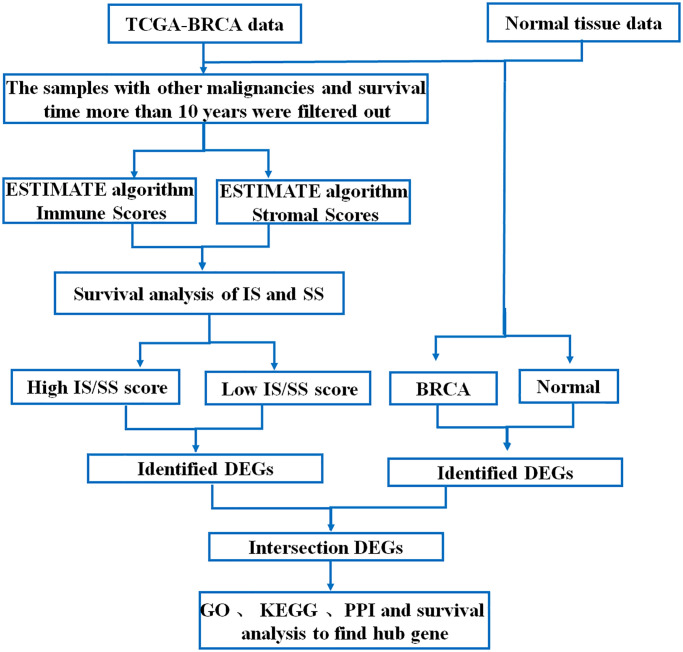
Flow chart of data processing in this study.

968 cases of breast cancer data were downloaded from TCGA-BRCA database, including mRNA sequencing data and corresponding clinical information. The data analysis scheme of this study is shown in Figure 1.

### Low immune score is associated with poor prognosis in Breast invasive carcinoma

**Figure 2:**
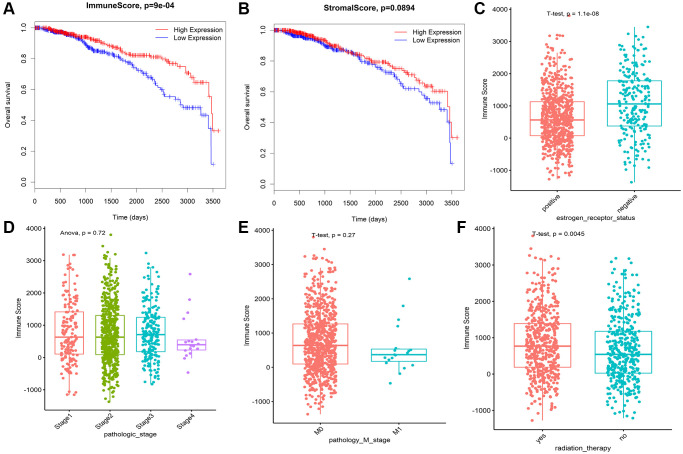
**Immune conditions are associated with BRCA overall survival and clinical features.** (**A**) Prognostic analysis of patients with differences in immune scores. (**B**) Prognostic analysis of patients with differences in stromal scores. (**C**) Correlation analysis between immune score and ER status of breast cancer. (**D**) The correlation between immune score and pathological stage of breast cancer was analyzed. (**E**) The correlation between immune score and M stage of breast cancer was analyzed. (**F**) The immune score was correlated with radiotherapy for breast cancer.

Immune score (IS) and stromal score (SS) in each 968 BRCA patients with complete clinical data were evaluated by ESTIMATE algorithm. BRCA patients were assigned to the high and low ARE/SS group, according to the median value of immune scores or stromal scores respectively. Kaplan-Meier curves showed that high immune score correlated with improved overall survival (OS) for BRCA (Figure 2A), while higher stromal score showed no significant benefits in OS (Figure 2B). Therefore, we focus on the genes associated with breast cancer prognosis and immune score. In addition, the immune scores were significantly associated with the subtype classification, including estrogen receptor status (Figure 2C), pathologic stage (Figure 2D), pathology M stage (Figure 2E), radiation therapy (Figure 2F).

### Composition of immune infiltration in breast invasive carcinoma patients

**Figure 3:**
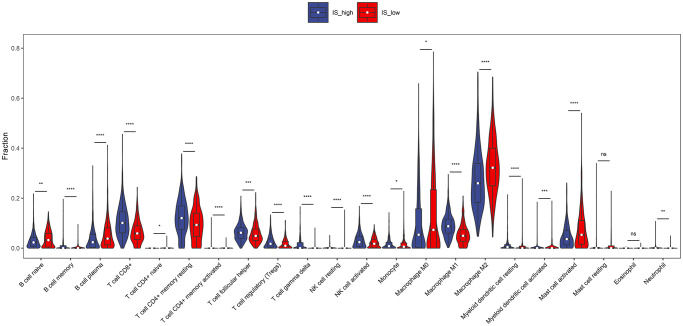
**The proportions of TIICs between high immune score group and low immune score group.** Difference of immune cell concentration between low-risk group and high-risk group. Red represents high-risk group while blue represents low-risk group.

**Figure 4:**
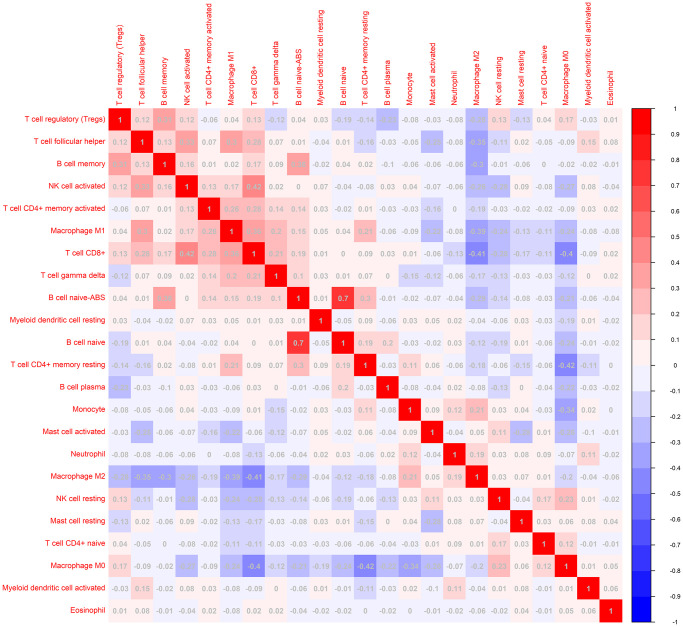
**The correlation matrix of 23 kinds of TLLs for breast cancer samples.** The relationship between the abundance ratios of various immune cells. The value represents the correlation value. Red represents a positive correlation, and the blue represents a negative correlation.

To investigate the landscape of immune infiltration in breast invasive carcinoma (BRCA), we estimated the immune infiltration level of each immune cell. The most important tumor-infiltrating lymphocytes (TIL) in breast cancer is macrophages, Mast cells activated and T lymphocytes. The higher proportion of B cell naïve, B cell plasma, Macrophage M0, Macrophage M2, Mast cells activated infiltration were found in low immune score group compared with the high immune score group, whereas proportion of T cell CD8^+^, T cell CD4^+^ memory resting, T cell follicular helper, Tregs, NK cell activated, Macrophage M1 cell infiltration were significantly higher in high immune score group (Figure 3). In addition, the correlation of 23 different TILs subsets was analyzed shown by the correlation heatmap (Figure 4). The populations with a significantly negative relation are Macrophage M0 and T cell CD4 memory resting (−0.42), Macrophage M2 and T cell CD8 (−0.41), Macrophage M0 and T cell CD4 (−0.4). The populations with a significantly positive relation were B cell naïve and B cell naïve-ABS (0.7); NK cells activated and T cell CD8; B cell naïve-ABS and B cell memory (0.36). These results suggest that the survival time of patients with high and low immune infiltration is significantly different.

### Identifying breast invasive carcinoma survival-related immune cells

**Figure 5:**
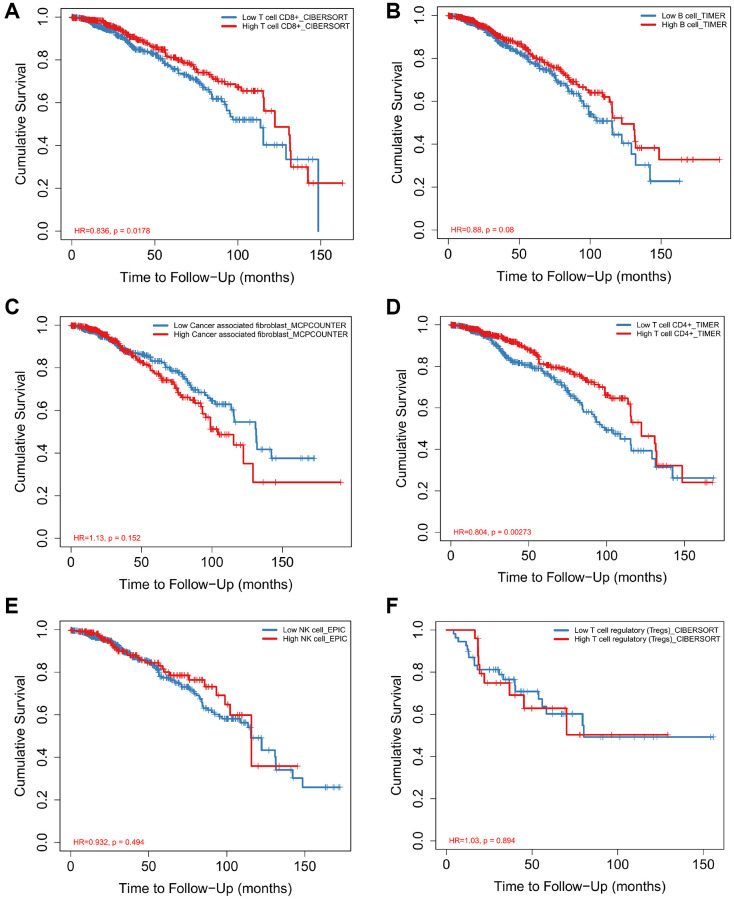
Analysis of immune cell proportion and survival time of breast cancer Survival curves for Macrophage M1 (**A**), Macrophage M2 (**B**), T cell CD8+ (**C**), B cell plasma (**D**), T cell follicular helper (**E**), T cell CD4+ memory activated (**F**) and prognosis of breast cancer were analyzed.

According to the data of CIBERSORTx 22 kinds of immune cell infiltration degree, the samples were divided into high infiltration and low infiltration according to the median value of infiltration degree. Combined with the survival time of the samples, the survival analysis was carried out to find the immune cells with significant effect on survival and survival. Macrophage M1 and T cell CD8 cells were positively related to 5-year overall survival in patients with BRCA (Figure 5A–5C). However, Macrophage M2 was negatively related to 5-year overall survival (Figure 5B). There was no significant correlation between B cell plasma, T cell CD4+ memory activated, T cell follicular helper and the 5-year survival rate of breast cancer (Figure 5D–5F). It is worth noting that although the remaining immune cells are not statistically significant, there is a clear trend.

### Differently expressed genes in high and low IS of breast invasive carcinoma

**Figure 6:**
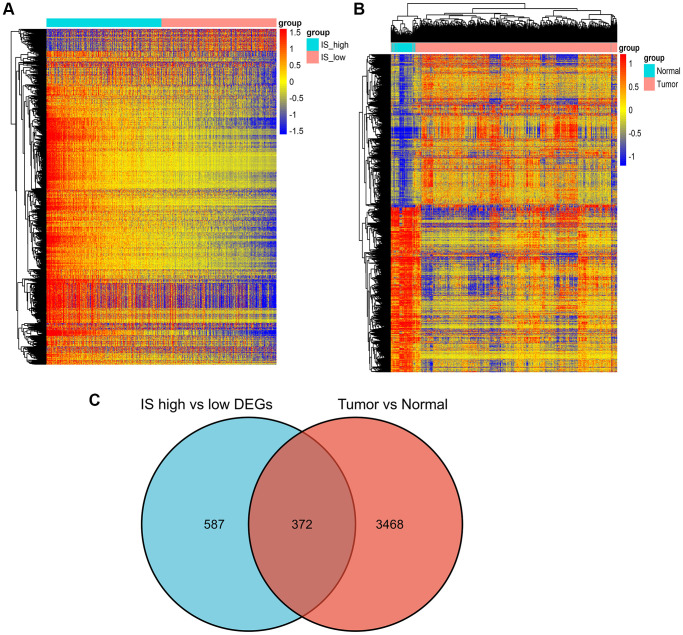
**Screening of differently expressed genes.** (**A**) Heatmap of DEGs for the high/low immune score groups. (**B**) Heatmap of DEGs for the BRCA/normal groups. (**C**) Commonly changed DEGs in the immune score and BRCA groups.

Differential gene expression was screened between the breast cancer high immune score group and the low immune score group with a difference of 2 and a significant threshold of P less than 0.05. A total of 959 differentially expressed genes were screened, including 659 up-regulated genes and 300 down regulated genes. (Figure 6A). Similarly, 900 significant differentially expressed genes were screened from breast cancer and normal tissues, of which 600 were up-regulated and 300 were down regulated (Figure 6B).

We identified 372 commonly differently expressed genes from the immune score and tumor groups (Figure 6C).

### Enrichment analysis of immune-related genes

**Figure 7:**
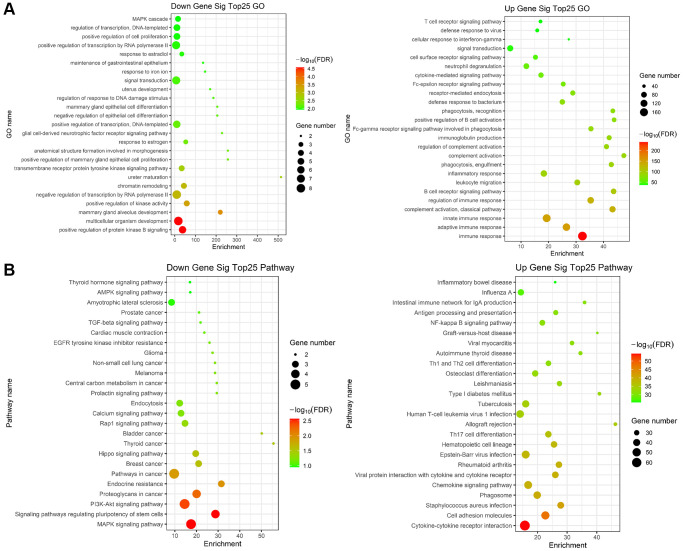
**Gene ontology (GO) and KEGG term enrichment analysis of common DEGs.** (**A**) The top 25 significantly enriched GO terms of down (left)/up (right) regulated genes. (**B**) The top 25 significantly KEGG of down (left)/up (right) regulated genes.

Subsequently, GO and KEGG enrichment analysis was performed on 372 differentially expressed genes. GO pathway enrichment showed those genes mainly enriched on immune response, adaptive immune response, innate immune response, B cell receptor signaling pathway, leukocyte migration, and inflammatory response (Figure 7A). We performed KEGG pathway enrichment and interrelation analysis. As shown in Figure 7B, enrichment of DEGs was mainly observed for the Cytokine-cytokine receptor interaction, Cell adhesion molecules, Staphylococcus aureus infection, Chemokine signaling pathway, Viral protein interaction with cytokine and cytokine receptor, Rheumatoid arthritis, Hematopoietic cell lineage.

### Protein-protein interaction (PPI) network construction and GSEA enrichment of hub genes

**Figure 8:**
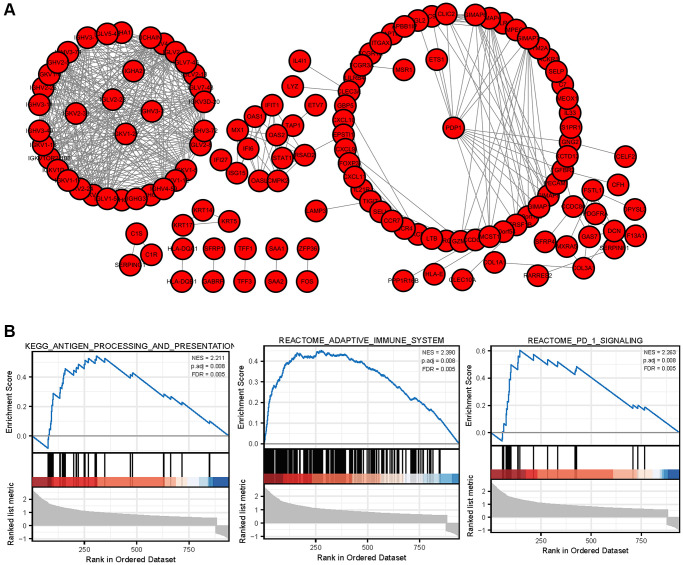
PPI network of DEGs and GSEA enrichment of hub genes (**A**) The protein interaction network of immune related differentially expressed genes in breast cancer. (**B**) GSEA enrichment analysis of breast cancer immune related core genes.

In order to further explore the interaction relationship of significant difference genes, the protein interaction network was constructed by using Cytoscape software. The protein interaction network of immune related differentially expressed genes is shown in Figure 8, which contains 75 nodes and 579 edges. The biological functions of immune related core differentially expressed genes were analyzed by GSEA. The results showed that immune related core differentially expressed genes were involved in response_ PD_ 1_ SIGNALING, REACTOME_ ADAPTIVE_ IMMUNE_ System and other biological processes.

### Survival analysis of hub genes in breast invasive carcinoma

**Figure 9:**
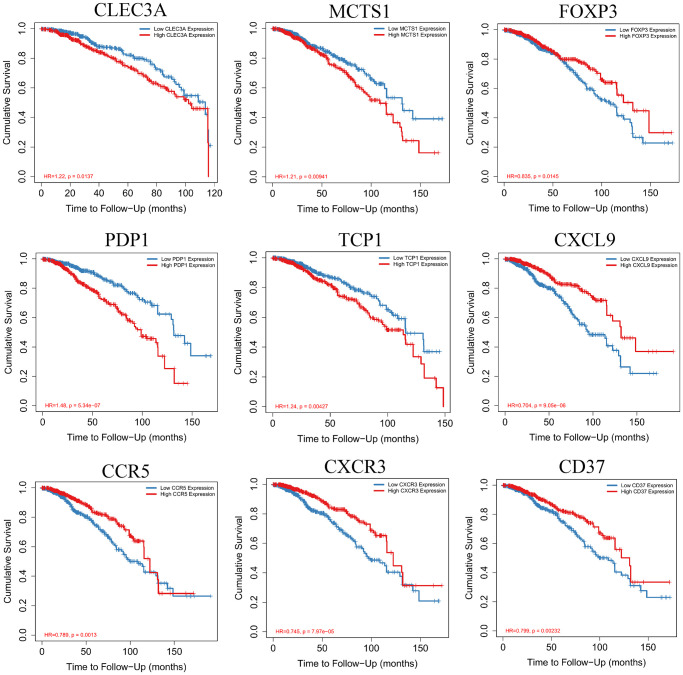
Kaplan-Meier survival curves with the log-rank test were performed for the hub genes.

Kaplan-Meier survival curves were constructed to determine the potential value of hub genes in predicting the overall survival of BRCA. Nine genes including CLEC3A, MCTS1, FOXP3, PDP1, TCP1, CXCL9, CCR5, CXCR3 and CD37 were closely related to the overall survival of BRCA. We also observed that the low expression of four genes (CLEC3A, MCTS1, PDP1 and TCP1,) was related to favorable survival outcomes. High expression of FOXP3, CXCL9, CCR5, CXCR3, and CD37 was related to a high overall survival rate in BRCA (Figure 9).

### The high expression of PDP1 in breast invasive carcinoma and correlation with clinical stage

**Figure 10:**
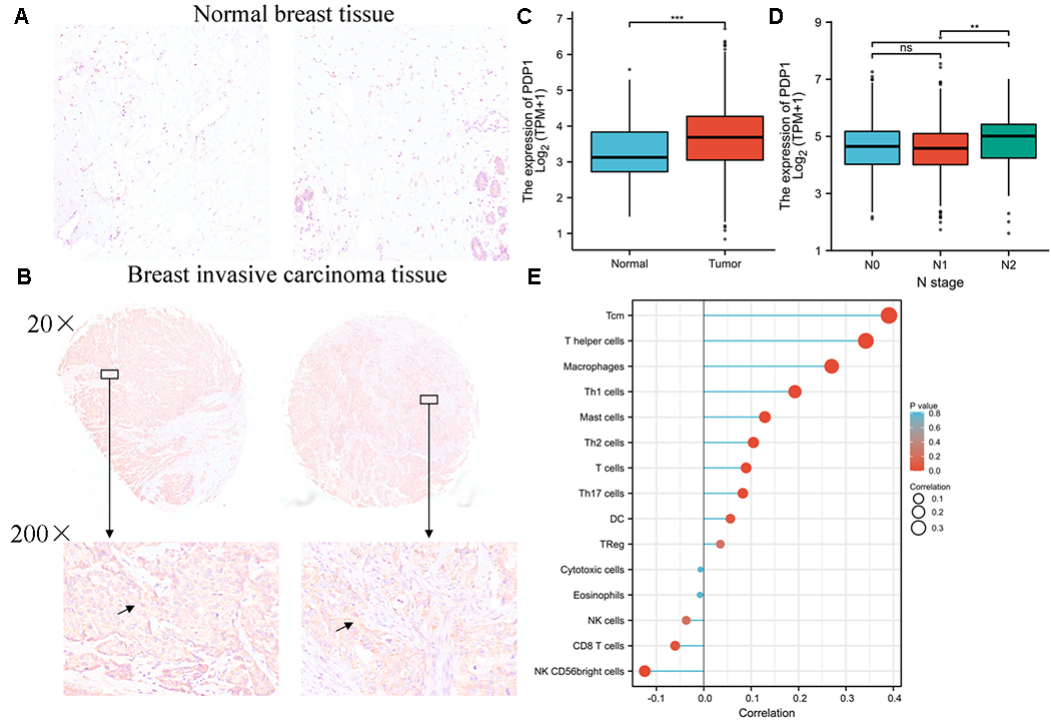
**PDP1 was up-regulated in breast invasive carcinoma.** (**A**) The level of PDP1 in normal tissues (*n* = 20) and (**B**) breast invasive carcinoma tissues (*n* = 70) was evaluated by immunohistochemical staining. The magnification is 100×. (**C**) The expression of PDP1 in BRCA tissues and normal tissues was analyzed by the cancer genome atlas (TCGA)-BRCA database. (**D**) The relationship between PDP1 expression and clinical N stage of breast cancer. (**E**) The relationship between PDP1 expression and breast cancer immune cell infiltration.

To investigate the discrimination of PDP1 expression in breast invasive carcinoma tissues, the expression of PDP1 in BRCA and normal tissues by immunohistochemistry were performed. We detected the expression of PDP1 in 70 cases of breast cancer and 20 cases of adjacent tissues by Immunohistochemical staining. The result indicates that PDP1 expression is higher in BRCA tissues (Figure 10A and 10B) compared to non-cancerous tissues. We analyzed the expression of PDP1 in BRCA tissues and normal tissues by bioinformatics, and the result of bioinformatics analysis indicated that PDP1 level was higher in BRCA tissues than that in normal tissues (Figure 10C). Moreover, clinicopathological parameters from BRCA patients were extracted, and we found that highly expressed PDP1 was positively correlated with N stage of BRCA patients (Figure 10D). It was found that the high expression of PDP1 was negatively correlated with the invasion of CD8T cells in breast cancer (Figure 10E).

## DISCUSSION

Tumor immune microenvironment, including anti-tumor immune effecter cells and molecules and immunosuppressive cells and molecules, plays an important role in the occurrence, development and clinical outcome of tumor [19–21]. In the process of tumorigenesis and development, the immune system mainly goes through the following three processes: immune surveillance, immune balance and immune destruction [9, 22–24]. Immune cells not only play a natural anti-tumor role at the beginning of tumor invasion, but also become a tumor promoting phenotype in the process of tumor progression, assisting tumor immune escape and distant metastasis. At present, tumor microenvironment immune characteristics have been listed as one of the top ten characteristics of tumor; On the other hand, it may play a role in predicting the efficacy of chemoradiotherapy [25–27]. Therefore, it is of great clinical significance to analyze the types and distribution of immune cells in tumor microenvironment and establish an effective immune evaluation system. Stromal cells and immune cells in tumor tissue constitute the main components of the dynamic network of tumor microenvironment [28–30]. This paper focuses on the analysis of the state of tumor microenvironment of breast cancer, and explores new ideas for prognosis evaluation and treatment strategies of breast cancer. ESTIMATE (estimation of stromal and immune cells in malignant tumor tissues using expression data) algorithm is an important tool to predict the purity of tumor and the proportion of stromal/immune cells infiltrated in tumor tissues [31–33]. It is mainly based on the gene expression profile data of tumor tissues from TCGA database.

The infiltration of different types of immune cells in the microenvironment of breast cancer and its role in prognosis prediction are not yet clear. We used the CIBERSORT algorithm to analyze the gene expression of breast cancer tissue and analyzed the correlation between the different types of immune cell infiltration and the overall survival of breast cancer patients. We demonstrated that Macrophage M1 and T cell CD8 cells were positively related to 5-year overall survival in patients with BRCA. However, Macrophage M2 was negatively related to 5-year overall survival. There was no significant correlation between B cell plasma, T cell CD4+ memory activated, T cell follicular helper and the 5-year survival rate of breast cancer.

Tumor infiltrating lymphocytes (TILs) are a group of heterogeneous lymphocytes around tumors. A large number of studies believe that TILs are the markers of the host immune system’s immune response to tumor antigens. Gu-Trantien et al. [34] classified breast tumor infiltrating immune cells in detail, in which T lymphocytes accounted for about 75%, B lymphocytes accounted for less than 20%, monocytes accounted for about 10%, while NK cells and NK/T cells accounted for less than 5%. TILs can be divided into stromal tumor infiltrating lymphocytes (sTILs) and intraepithelial tumor infiltrating lymphocytes (iTILs). The former is located in the tumor stroma without direct contact with invasive cancer cells, while the latter is intraepithelial or intratumoral infiltrating cells in direct contact with cancer cells.

Furthermore, we analyzed the relationship between the breast cancer microenvironment and the prognosis of breast cancer patients. We found that the patients with high immune scores in the tumor microenvironment had a better prognosis than those with immune score. By analyzing and comparing the gene expression profiles of two groups with high and low score of breast cancer, we screened 1367 differentially expressed genes. Among them, 401 differentially expressed genes were significantly correlated with the prognosis of breast cancer patients. By constructing the protein interaction network and mining its core modules, we finally obtained 79 core differentially expressed genes associated with prognosis, and further verified 9 core prognostic differentially expressed genes (CLEC3A, MCTS1, FOXP3, PDP1, TCP1, CXCL9, CCR5, CXCR3 and CD37). The results showed that the low expression of four genes (CLEC3A, MCTS1, PDP1 and TCP1,) was related to favorable survival outcomes. High expression of FOXP3, CXCL9, CCR5, CXCR3, and CD37 was related to a high overall survival rate in BRCA.

In conclusion, this study used bioinformatics methods to screen and analyze differentially expressed genes that might be related to the immune microenvironment and prognosis of breast cancer, to identify potential regulatory mechanisms, and to predict potential therapeutic agents, so as to provide effective support for the prognosis evaluation and individualized treatment strategies of breast cancer.

## MATERIALS AND METHODS

### TCGA-BRCA data downloading and processing

The raw data and clinical information were downloaded from the TCGA-BRCA project (https://cancergenome.nih.gov/). After removing the samples that had suffered from other malignant tumors and survived for more than 10 years, the final samples used in this study were tumor 968 cases and normal 106 cases.

### Immune score and stromal score analysis

The immune infiltration status of each BRCA patient were determined by applying the the R package ESTIMATE algorithm. Briefly, the estimate algorithm is a tool to predict the proportion of stromal cells and immune cells in tumor tissue by using gene expression characteristics.

### Identification of differential expressed genes with immune scores

BRCA patients were divided into high- and low immune score groups according to their median of immune score. Briefly, Deseq2 was applied to identify DEGs by comparing high/low IS or BRCA and normal samples. The Benjamini-Hochberg method was used to adjust the *p*-value. A gene with false discovery rate (FDR) adjusted *p*-value <0.05 and |log2FC|>1 is identified as DEG.

### GO and KEGG enrichment analysis

To explore the function of DEGs regulated by methylation in the carcinogenesis and development of colon cancer, GO enrichment analysis was performed using DAVID database (https://david.ncifcrf.gov/) and three categories: cellular component (CC), molecular function (MF), and biological process (BP) were analyzed. In addition, KEGG pathways were analyzed using KEGG Orthology-Based Annotation System 3.0.

### Survival-related immune cells identification

Immune infiltration of each patient was calculated by R package CIBERSORT. CIBERSORT algorithm was used to analysis 22 types of immune cells. Correlation between immune cell abundance was processed using Pearson’s correlation coefficient. Correlation between gene and immune cells abundance was also estimated by Pearson’s correlation coefficient.

### Analysis of the expression level of PDP1 in BRCA and normal tissues

The Oncomine database was used to identify the expression level of PDP1 in various types of tumor tissues. Screening conditions: *P* < 0.05, multiple of difference >1.5, the top 10% of genes are ranked, and the data type is mRNA. The expression level of PDP1 in BRCA and normal tissues in the TCGA database was compare and analyze.

### PDP1 immunohistochemical staining

Tissue microarray containing 70 cases of breast cancer and 20 tissues was purchased from Shanghai OUTDO BIOTECH Company. After dewaxing and hydration, the tissue sections were sealed with goat serum at room temperature for 2 hours. Tissues section were followed by incubation with primary antibody against PDP1 (21176-1-AP, 1:100, Proteintech) for 12 h at 4°C. Thereafter, the sections were incubated with biotin labeled Goat anti rabbit IgG polymer and horseradish enzyme labeled Streptomyces ovalbumin working solution for 1 h. After that, the slides were then stained by 3, 3’-Diaminobenzidine (DAB) solution (Sigma-Aldrich) and subsequently counterstained with hematoxylin (Sigma-Aldrich). Finally, of immunohistochemical staining were obtained using Aperio Scanscope slide scanner (Leica, GT450).

### Statistical analysis

Data were analyzed using R package. Data were represented as mean ± standard deviation (S.D.). All tests were two sided, and *P* < 0.05 was considered statistically significant.
